# Chelidonic acid abrogates oxidative stress and memory dysfunction in experimental aging rats

**DOI:** 10.55730/1300-0152.2717

**Published:** 2024-10-30

**Authors:** Feride Nihal SİNAN, Emel SERDAROĞLU KAŞIKÇI, Burcu ÇEVRELİ

**Affiliations:** 1 Graduate School of Science, Department of Biotechnology, Üsküdar University, İstanbul, Turkiye; 2 Neuropyschopharmacology Application and Research Center, Üsküdar University, İstanbul, Turkiye; 3 Department of Molecular Biology and Genetics, Faculty of Engineering and Natural Sciences, Üsküdar University, İstanbul, Turkiye

**Keywords:** Aging, chelidonic acid, D-galactose, antioxidant, memory

## Abstract

**Background/aim:**

In an aging model established using male Wistar albino rats via the administration of D-galactose (D-gal), the aim of this study was to examine the effects of chelidonic acid (CA) on cognitive function and the levels of glutathione (GSH), malondialdehyde (MDA), total antioxidant status (TAS), and brain-derived neurotrophic factor (BDNF).

**Materials and methods:**

Thirty-two, three-month-old Wistar albino male rats (n = 8) were divided into four groups, as the control (C) group, CA group (2 mg/kg of CA via oral gavage), D-gal group (150 mg/kg of D-gal, subcutaneously), and D-gal + CA group (150 mg/kg of D-gal and 2 mg/kg of CA). Following overnight fasting, the 10-week trial was concluded with intramuscular injections of anesthetic drugs xylazine (8–10 mg/kg) and ketamine (80–100 mg/kg), and subsequently, the collection of cardiac blood. The brain tissues of the rats were removed. The GSH, MDA, TAS, and BDNF levels were determined in the collected serum samples and prepared tissue homogenates. Novel object recognition and Morris water maze (MWM) experiments were also used to evaluate cognitive function.

**Results:**

The D-gal group demonstrated a statistically significant improvement in the discrimination index for memory in both the short and long term compared to the D-gal + CA group. Further analysis of the MWM data for these two groups indicated a notable decrease in the amount of time required for finding the platform. In comparison with the D-gal group, the MDA levels decreased in the CA and D-gal + CA groups, whereas the GSH, TAS, and BDNF levels increased in both the serum and hippocampus samples.

**Conclusion:**

CA showed positive effects on age-related neurodegenerative disorders and memory-related processes, especially by increasing TAS and BDNF levels.

## 1. Introduction

Aging is characterized by the progressive accumulation of physiological changes, resulting in a decline in functional capacities over time (Azman and Zakaria, 2019). It represents a biological phenomenon leading to the gradual deterioration of physiological functions, thereby contributing to increased rates of morbidity and mortality. Brain decline, which includes dementia, cognitive impairment, and progressive loss of memory and learning, is mostly caused by aging. Elevated concentrations of reactive oxygen species (ROS) have the potential to induce oxidative stress and harm proteins, phospholipids, and DNA structure, which can eventually result in damage to cells and tissues. This is a key process for aging brought on by ROS (Sun et al., 2018). Although there are studies showing that antioxidants reduce malondialdehyde (MDA) levels, the amount of antioxidants that must be consumed to show these positive effects remains unclear. For this reason, the total antioxidant status (TAS) is a frequently used parameter in the measurement of the activity of all antioxidants together. Because the separate measurement of different oxidant and antioxidant molecules is impractical and time-consuming (Can et al., 2016; Demircigil et al., 2023).

The body naturally contains D-galactose (D-gal) (Lee et al., 2020). Changes that mimic aging are brought about by long-term D-gal treatment. Galactokinase and uridyl transferase convert D-gal into glucose via metabolization. The overabundance of D-gal accumulates through several metabolic pathways, forming galactitol and galactonate, which cause osmotic stress and increased ROS generation. Age-related changes in many organs, including the heart, kidneys, and brain, are mediated in large part by oxidative damage and inflammation. D-gal-induced aging serves as a well-established experimental model in rodents, offering a platform to investigate the aging process and potential pharmacological interventions devoid of the accompanying comorbidities seen in natural aging. The brain, characterized by its elevated lipid content and oxygen consumption, is notably susceptible to oxidative stress (Sumbalová et al., 2022). Neurological deficits, reduced antioxidant activity, and increased neuroinflammation are all significantly correlated with aging (Lian et al., 2017; Liu et al., 2019; Hong et al., 2023). Cognitive loss throughout aging is caused by a decrease in brain-derived neurotrophic factor (BDNF) and the impairment of astrocyte function (Hong et al., 2023).

BDNF, a neuron cell growth protein, increases neurogenesis in the nervous system by improving synaptic functioning and cell survival. Neuroprotection against neuronal cell death is facilitated by the activation of extracellular signal-regulated protein kinase triggered by the interaction of BDNF receptors. According to Jeong et al. (2016), synaptogenesis, neurogenesis, synaptic plasticity, and cell survival are among the processes that support memory and learning that are significantly regulated by BDNF. Decreased expression of BDNF and dysregulated gamma-aminobutyric acid (GABA) neurotransmission are evident in psychiatric disorders such as depression, schizophrenia, and during the aging process (Tomoda et al., 2022). GABA_B_ autoreceptors and BDNF are important for the establishment of GABAergic synapses and GABA_A_ receptor-mediated transmissions in the developing rat hippocampal regions (Mizoguchi et al., 2006).

Chelidonic acid (CA) is an organic acid that is heterocyclic and has a pyran skeleton. It is a constituent of *Chelidonium majus L*. According to Jeong et al. (2016), it inhibits the enzyme glutamate decarboxylase in the rat brain and possesses neurological sedative, moderate analgesic, antiallergic, and antiulcerative colitis properties. According to Singh et al. (2016), CA is a strong inhibitor of glutamic acid decarboxylase. As a result, it is crucial for the synthesis of GABA.

Studies have reported that CA is an immunomodulator, neurotransmitter regulator, anticancer agent, and histamine release inhibitor in rat peritoneal mast cells, reduces TNF-α, is a mild analgesic, and a competitive inhibitor of the central nervous system (Porter and Martin, 1985; Shin et al., 2011; Avdeeva et al., 2019; Hamdi et al., 2023). Considering all these effects of CA, it is seen as an alternative compound for treatment (Miroshnichenko et al., 2022). However, while there are studies on the antioxidant effects of CA in the literature, its effectiveness in an aging model has not been evaluated. Therefore, herein, it was aimed to investigate the preventive treatment potential of CA on oxidative stress, learning, and memory in an aging model that also causes neurodegenerative disorders such as Alzheimer’s disease (AD).

## 2. Materials and methods

### 2.1. Animals

This study was carried out at the Üsküdar University Experimental Research Unit (ÜSKÜDAB) laboratory. Thirty-two, three-month-old, male Wistar albino rats were housed in standard cages at 22 + 2 °C, under a light/dark photoperiod of 12:12, and fed standard pellet feed ad libitum.

### 2.2. Experimental procedure

**Figure 1:**
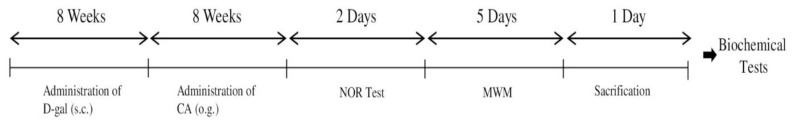
Experimental chronology.

The rats were divided into four groups, as the control (C) group; D-gal group: the rats were given D-gal (150 mg/kg/day, subcutaneously) dissolved in 0.5 mL of saline (Liao et al., 2023) for eight weeks; CA group: the rats received oral CA at a dose of 2 mg/kg/day in saline (Jeong et al., 2016) for eight weeks; and D-gal + CA group: the rats received oral CA at a dosage of 2 mg/kg/day after receiving D-gal (150 mg/kg/day, intraperitoneally) for eight weeks (Figure 1).

### 2.3. Novel object recognition (NOR) test

**Figure 2:**
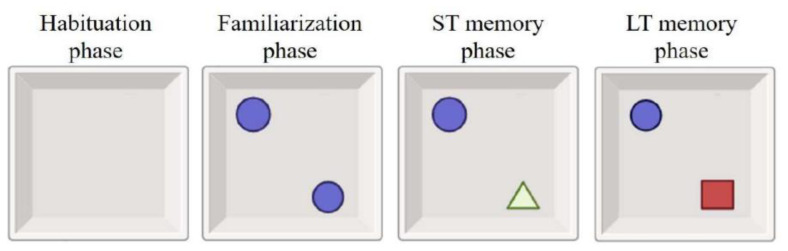
NOR test experimental setup (Bitmez et al., 2024).

The NOR test is a popular multipurpose assessment tool for short- and long-term memory that rates recognition-based memory and exercise activities (Grayson et al., 2015). The test is based on the properties of objects before they are introduced to rats, rather than objects that have been previously introduced (Turan et al., 2023). The test is administered in a maze monitored by a camera. During the practice period, the rats are given 10 min to familiarize themselves with two unique objects. To be exact, each of the objects is swapped out for another, and the rats are allowed to concentrate on the new object for 5 min. Then, the newly placed object is replaced with a different object, and the measurement is made in the same way (Figure 2). The results obtained are then calculated and analyzed as discrimination and recognition indices (Küçükkarapinar et al., 2021). The following method was used in the calculation of the discrimination index (Koca, 2019).


Discrimination index=(new object-familiar object)/(new object+familiar object)

### 2.4. Morris water maze (MWM)

The MWM is used to measure and evaluate learning and cognitive activity. In addition to cognitive parameters, it is used to evaluate delay and short- and long-term memories (Wu et al., 2021). The MWM is commonly employed in controls and comparisons of in vivo models that disrupt or affect hippocampal activities in rats (Lissner et al., 2021). Moreover, it was employed by Li et al. (2022) in a D-gal-induced aging model to evaluate memory and learning. One of the best assessments for assessing learning and memory abilities, particularly those that depend on the hippocampus, is the MWM. The learning phase involves repeatedly teaching the location of the hidden platform. This is followed by the testing phase, where the previously taught position of the hidden platform is expected to be found. The learning process is aided by placing guiding cues around the tank (Babur et al., 2017). The tank, which has a diameter of 150 cm and a height of 60 cm, is filled with water. A camera system placed above the tank transmits the image to a monitor, and measurements are made via video (SMART 3.0). The resulting image is divided into four different quadrants (northwest, northeast, southwest, and southeast), and the platform is placed in a randomly selected quadrant. For four days, rats are released into the water from designated quadrants and given 120 s to find the platform. For 15 s, rats that are unable to locate the platform are led to it and left there. On day five, the rats are expected to navigate to the area where the platform was previously located (Topuz, 2015).

### 2.5. Preparation of the serum and brain tissue samples

The rats were anesthetized with xylazine-ketamine before the blood samples were taken at the conclusion of the experiment, following 12 h of fasting. The samples were centrifuged for 10 min at 3000 rpm. After that, the collected brain tissues and serum samples were stored at –80 °C for subsequent use. The brain tissues were first washed with physiological saline (NaCl) and then homogenized under cold ice.

### 2.6. Determination of the glutathione (GSH) levels

Serum and homogenized brain tissue GSH concentrations were ascertained following the method by Beutler et al. (1963) employing 5,5′-dithiobis (2-nitrobenzoic acid) for color development and metaphosphoric acid for protein precipitation.

### 2.7. Determination of the MDA levels

The MDA levels in the serum and homogenized brain tissue samples were evaluated based on the investigation of lipid peroxidation (LPO) levels. In accordance with the method of Çoban et al. (2015), the LPO level was determined by measuring the concentration of MDA using thiobarbituric acid.

### 2.8. ELISA assays

The TAS and BDNF level were measured using kits from Rel Assay Diagnostics (Gaziantep, Turkey) and Shanghai Sunred Biological Technology Co., Ltd. (Baoshan District, Shanghai, China).

### 2.9. Statistical analysis

GraphPad Prism 9 (GraphPad Software, San Diego, CA) was utilized for the statistical analysis. p < 0.05 was accepted as statistically significant for comparisons between the groups using one-way ANOVA and the LSD test. The results were displayed as the mean ± standard error of the mean.

## 3. Results

### 3.1. Comparison of recognition memory performance between the groups

**Figure 3:**
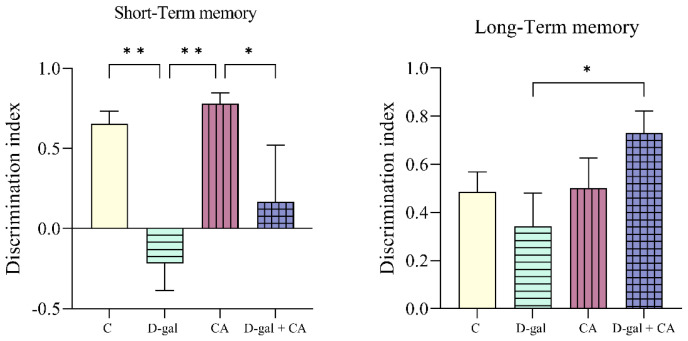
Evaluation of both short-term and long-term memory (* p < 0.05 and ** p < 0.01).

When comparing the C group with the D-gal group, the discrimination index for short-term memory was lower in the D-gal group (p = 0.0065). When comparing the CA group with the D-gal and D-gal + CA groups, the discrimination index of the D-gal (p = 0.0024) and D-gal + CA (p = 0.0452) groups was lower. For long-term memory, when compared with the D-gal group, the discrimination index of the D-gal + CA group was higher (p = 0.0239) (Figure 3).

### 3.2. Comparison of spatial memory performance between the groups

**Figure 4:**
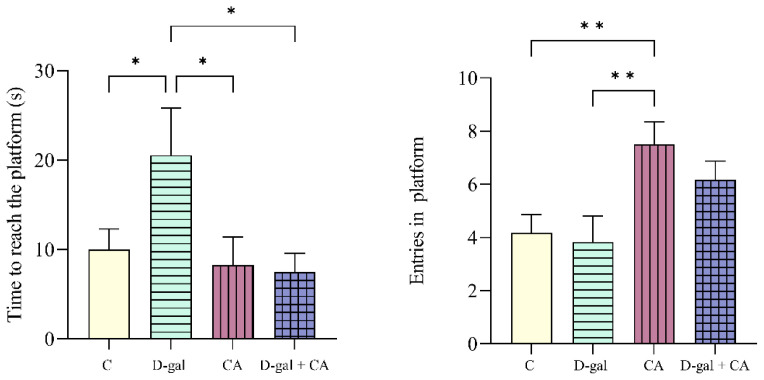
Latency time and entries into the platform zone (* p < 0.05 and ** p < 0.01).

When compared with the D-gal group, the C (p = 0.0428), CA (p = 0.0202), and D-gal + CA (p = 0.0142) groups reached the platform in a shorter time. When compared with the CA group, the C (p = 0.0091) and D-gal (p = 0.0048) groups had lower numbers of entries to the platform (Figure 4).

### 3.3. Comparison of the GSH-serum, GSH-hippocampus, MDA- serum, MDA-hippocampus parameters of the groups

**Figure 5:**
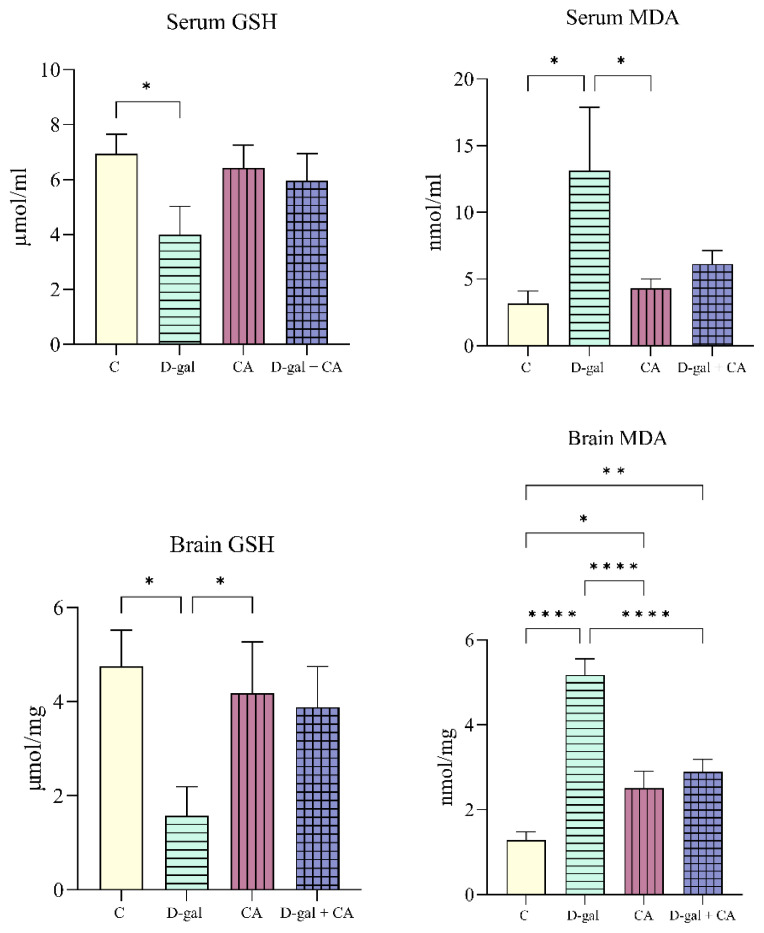
Serum and hippocampus GSH and MDA levels (* p < 0.05, ** p < 0.01, and **** p < 0.0001).

When compared to the C group, the serum GSH level was lower in the D-gal group (p = 0.0325). When compared to the D-gal group, the serum MDA levels were lower in the C (p = 0.0102) and CA (p = 0.0204) groups. When compared to the D-gal group, the hippocampus GSH levels were higher in the C (p = 0.0153) and CA (p = 0.0423) groups. When compared to the D-gal group, the hippocampus MDA levels were lower in the C, CA, and D-gal + CA (p < 0.0001) groups. Compared to the C group, the MDA levels were higher in the CA (p = 0.0161) and D-gal + CA (p = 0.0026) groups (Figure 5).

### 3.4. Comparison of the TAS and BDNF levels of the groups

**Figure 6:**
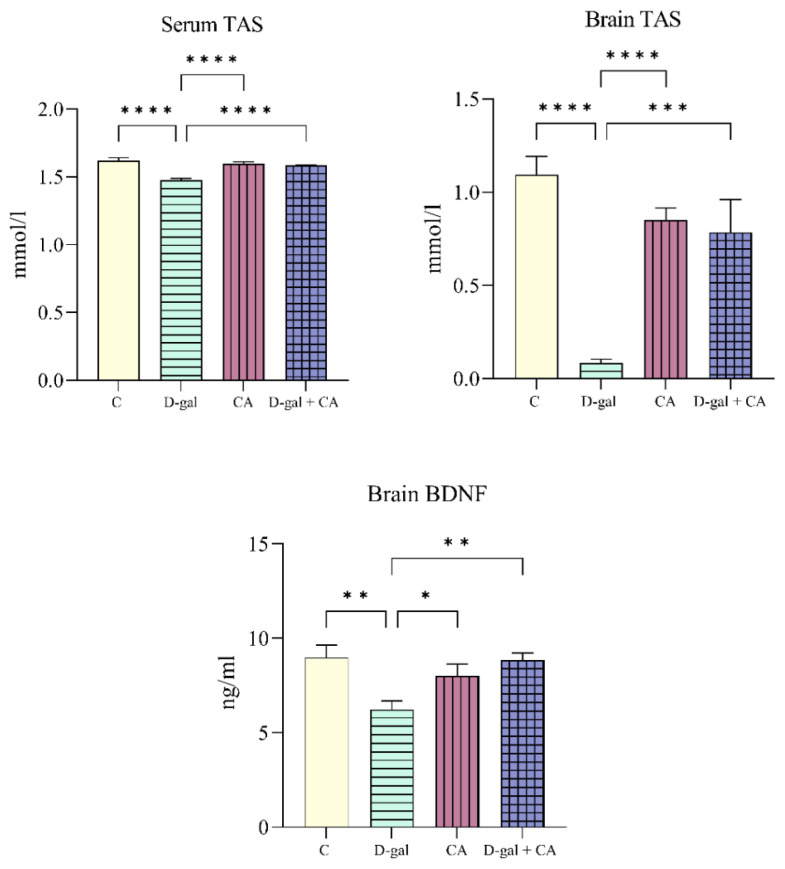
Serum and hippocampus TAS levels and hippocampus BDNF levels (* p < 0.05, ** p < 0.01, *** p < 0.001, and **** p < 0.0001).

When compared to the C groups, the serum TAS levels were higher in the CA (p = 0.0185) and D-gal + CA (p = 0.011) groups. When compared to the D-gal group, the serum TAS levels were higher in the CA (p = 0.0263) and DC (p = 0.0158) groups. When compared to the D-gal group, the hippocampus TAS levels were higher in the C (p < 0.0001), CA (p = 0.0004), and D-gal + CA (p = 0.0002) groups. When compared to the D-gal group, the hippocampus BDNF levels were higher in the C (p < 0.0001), CA (p < 0.0001), and D-gal + CA (p = 0.0040) groups. Additionally, when compared to the D-gal + CA group, the hippocampus BDNF levels were higher in the CA group (p = 0.0051) (Figure 6).

When the effects of CA on memory and neurological functions were examined, normalized short- and long-term and spatial memory function were damaged by D-gal. The D-gal group showed substantial changes in the TAS levels, which were restored to normal after receiving CA therapy. Additionally, the BDNF levels, which decreased in the D-gal group, normalized in the D-gal + CA group, indicating a positive effect of CA on neurological function.

## 4. Discussion

The effects of CA on cognitive function, and GSH, MDA, TAS, and BDNF levels were investigated using a natural aging model created using male Wistar-Albino rats treated with D-gal. The results were discussed in comparison with the literature.

Various studies have shown that aging models induced with different doses of D-gal exhibited lower discrimination indices in the NOR test compared to the C or treatment groups. For example, Fatemi et al. (2018) reported a decrease in the tendency of the D-gal group toward the novel object in their NOR test results. Sun et al. (2018) observed a decrease in the recognition index of the D-gal group compared to the C group in their NOR test after subcutaneous injections of 150 mg/kg for six weeks in Institute of Cancer Research mice. Gao et al. (2022) reported a decrease in the ability of D-gal-treated Kunming mice to discriminate compared to the C group in their NOR test after subcutaneous injections of 100 mg/kg for eight weeks. Similarly, Lei et al. (2023) noted a decrease in the recognition index of D-gal-treated Sprague-Dawley rats compared to the C group after subcutaneous injections of 125 mg/kg for eight weeks. Liao et al. (2023) and Dai et al. (2023) also reported reductions in the recognition index compared to the C group in their respective studies using D-gal-treated rats.

Herein, D-gal, which was used to mimic natural aging, impaired learning and reduced the discrimination index (scoring the ability to distinguish objects) to below zero in the NOR test. Additionally, although there was no statistically significant difference in the long-term memory evaluation, the discrimination index was observed to decrease in the C group when compared with the D-gal group. Thus, there were parameters supporting the establishment of the current model.

When short-term memory was examined, the discrimination index was higher in the CA group than in the C group, lower in the D-gal + CA group than in both the C and CA groups, but higher than in the D-gal group. Furthermore, when long-term memory was examined, the D-gal + CA group showed a higher discrimination index, indicating that CA may have the potential to support visual memory. It was reported that cholinergic deficiency in AD leads to short-term memory loss (Cummings et al., 2019; Lopez et al., 2019). AD is characterized by synaptic loss and the inability to form and store memories, which are clinical features of the disease. Postsynaptic receptors serve as a link between structural and functional plasticity and are essential for the formation of short-term memory. Dendritic spines are crucial for cognitive resilience. Changes in the number of synapses and postsynaptic receptors occur early on in AD, and these changes are associated with cognitive decline. The selective loss of postsynaptic receptors and the distinct mechanisms related to the storage of long-term memory might explain the relatively preserved formation of implicit memory, even in the later stages of the disease (Lopez et al., 2019). In light of this information, it can be suggested that short-term memory impairment is affected earlier and more significantly in diseases like AD, which manifest with aging. Despite the significant decline in short-term memory in the D-gal group, long-term memory was not significantly reduced, and thus, no complete deterioration was observed. Consequently, the D-gal + CA group showed better performance following CA treatment (Figure 3).

The D-gal group takes longer than the C or treatment groups to find the platform in MWM results, according to a number of studies looking at aging models created with varying doses of D-gal. According to Gao et al. (2022), cognitive impairment was indicated by an increase in the time it took to discover the platform and a decrease in the amount of time spent on and crossing over the platform quadrant. Lei et al. (2023) observed that the D-gal group had memory impairment as evidenced by longer platform finding times and fewer entries onto the platform than the C group. Dai et al. (2023) reported longer platform finding times, fewer crossings over the platform, and thus supported cognitive impairment. Rehman et al. (2017) observed an increase in the platform finding time, a decrease in learning and memory ability, and fewer entries onto the platform compared to treatment groups in their study of D-gal-treated Sprague-Dawley rats. Yuan et al. (2020) reported that the D-gal group took longer to find the platform compared to the treatment group. Khedr et al. (2022) noted a delay in reaching the platform in their study of D-gal-treated Wistar rats. Cui et al. (2023) observed longer platform finding times and fewer crossings over the platform in their study of D-gal-treated Kunming mice. In light of all this information, the delay in finding the platform in the D-gal group supports the formation of the current model.

When spatial memory was examined in the CA and D-gal + CA groups, the time to reach the platform supports the idea that CA may enhance memory. When comparing the number of crossings over the platform area, the CA and D-gal + CA groups made more crossings compared to the C and D-gal groups. These data implicate the positive effects of CA. At the same time, in the CA and D-gal + CA groups, there was no significant difference in the platform finding time compared to the C group, and the findings related to crossings over the platform area indicated that the CA and D-gal + CA groups demonstrated a consistent orientation toward the platform (Figure 4). The time it took for the CA and D-gal + CA groups to reach the platform supports the idea that CA may enhance memory. Furthermore, the current study is the first to examine the effect of CA on recognition and spatial memory.

It is known that D-gal induces biochemical abnormalities in the brain during aging, such as decreased GSH levels, increased MDA levels, decreased antioxidant enzyme activity, and the weakening of cholinergic neurons (Azman and Zakaria, 2019; Ameen et al., 2022). Rehman et al. (2017) reported a significant increase in brain MDA levels with D-gal administration. Sun et al. (2018) noted a decrease in the total antioxidant capacity (T-AOC) in the plasma and brain and an increase in MDA levels. Yuan et al. (2020) indicated an increase in serum MDA levels in the D-gal group, with decreased GPx and T-AOC activity. Li et al. (2021) reported increased serum and brain MDA levels and decreased T-AOC activity. Lin et al. (2021) reported increased brain MDA levels and decreased GSH levels and T-AOC activity in their study of Kunming mice. Daroi et al. (2022) found increased brain MDA levels and decreased GSH levels with D-gal administration. In comparison with the C group, Yue et al. (2023) observed elevated MDA levels in the serum and hippocampal regions following D-gal administration. Additionally, GSH levels were significantly higher in the C group but lower in the treatment groups. Liao et al. (2023) reported increased brain MDA levels and decreased GSH and BDNF levels determined by Western blot. Yin et al. (2024) reported increased MDA levels and decreased GSH levels in the hippocampus with D-gal administration. Fatemi et al. (2018) and Nam et al. (2019) observed significant decreases in brain BDNF levels with D-gal administration. Dai et al. (2023) found lower hippocampal BDNF levels with D-gal administration.

Based on the literature review, the data obtained in the present study, showing low GSH levels in the serum and hippocampus and high MDA levels in the D-gal group, biologically support the model herein as mimicking oxidative stress and aging. When examining the CA and D-gal + CA groups, although the GSH levels were higher and MDA levels were lower compared to the D-gal group, these groups had lower in GSH levels and higher in MDA levels compared to the C group. Khairnar et al. (2024), in their study using Wistar rats and the cancer preventive drug cisplatin, reported that CA reduced the nephrotoxicity of cisplatin, showing that the MDA levels, which were higher in the C group, were lower compared to the cisplatin group, and the GSH levels, which were lower in the C group, were higher compared to the cisplatin group, demonstrating that CA exhibited antioxidant effects in their model. In this context, the results of their study were also in parallel to the current findings (Figure 5). Additionally, the serum TAS levels were higher in the CA and D-gal + CA groups compared to the C and D-gal groups. These results largely support the antioxidant effect of CA. The lower hippocampus TAS levels in the D-gal group compared to all the other groups were a significant finding of the cognitive effect of CA. The hippocampus BDNF levels were lower in all the groups compared to the D-gal group (Figure 6).

It was concluded that CA may have favorable effects on cognitive function and antioxidant properties against oxidative stress in light of the available data. Thus, additional research is necessary to clarify CA, as its effects and mechanisms are unclear, through larger studies and various dose trials, as well as shed light on its potential.

## References

[b1-tjb-48-06-432] Ameen O Samaka RM Abo-Elsoud RA 2022 Metformin alleviates neurocognitive impairment in aging via activation of AMPK/BDNF/PI3K pathway Scientific Reports 12 1 17084 10.1038/s41598-022-20945-7 36224264 PMC9556637

[b2-tjb-48-06-432] Avdeeva E Shults E Rybalova T Reshetov Y Porokhova E 2019 Chelidonic acid and its derivatives from Saussurea Controversa: Isolation, structural elucidation and influence on the osteogenic differentiation of multipotent mesenchymal stromal cells in vitro Biomolecules 9 5 189 10.3390/biom9050189 31100934 PMC6572306

[b3-tjb-48-06-432] Azman KF Zakaria R 2019 D-Galactose-induced accelerated aging model: an overview Biogerontology 20 6 763 782 10.1007/s10522-019-09837-y 31538262

[b4-tjb-48-06-432] Babur E Tan B Yıldız N Batakçı M Süer C 2017 Gender-dependent differences in spatial learning performance in hypothyroid rats Journal of Health Sciences 26 3 233 239 (in Turkish)

[b5-tjb-48-06-432] Beutler E Duron O Kelly BM 1963 Improved method for the determination of blood glutathione The Journal of Laboratory and Clinical Medicine 61 882 888 13967893

[b6-tjb-48-06-432] Bitmez B Çevreli B Kaşıkçı E 2024 Effect of thymol on oxidative stress and reelin signaling pathway in Alzheimer’s disease model Turkish Journal of Biology 48 1 70 79 10.55730/1300-0152.2683 38665779 PMC11042864

[b7-tjb-48-06-432] Can Ü Yerlikaya FH Yener Y Çakır S 2016 Effects of High-Fat Diet and Acrylamide on Tissue Oxidant and Antioxidant Levels in Rats Selçuk Tıp Dergisi 32 2 38 42 (in Turkish with an abstract in English)

[b8-tjb-48-06-432] Cui B Liu L Shi T Yin M Feng X 2023 The Ethanolic Extract of Lycium ruthenicum Ameliorates Age- Related Physiological Damage in Mice Molecules 28 22 7615 10.3390/molecules28227615 38005337 PMC10673502

[b9-tjb-48-06-432] Cummings JL Tong G Ballard C 2019 Treatment combinations for Alzheimer’s disease: current and future pharmacotherapy options Journal of Alzheimer’s disease 67 3 779 794 10.3233/JAD-180766 PMC6398562 30689575

[b10-tjb-48-06-432] Çoban J Doğan Ekici I Aydın AF Betül Kalaz E Doğru Abbasoğlu S 2015 Blueberry treatment decreased D-galactoseinduced oxidative stress and brain damage in rats Metabolic Brain Disease 30 793 802 10.1007/s11011-014-9643-z 25511550

[b11-tjb-48-06-432] Dai XJ Jia Y Cao R Zhou MN 2023 Naringin prevents cognitive dysfunction in aging rats by inhibiting toll-like receptor 4 (TLR4)/NF-κB pathway and endoplasmic reticulum stress Evidence Based Complementary and Alternative Medicine 2919811 10.1155/2023/2919811 36865741 PMC9974290

[b12-tjb-48-06-432] Daroi PA Shrikant ND Archana RJ 2022 p-Coumaric acid protects against D-galactose induced neurotoxicity by attenuating neuroinflammation and apoptosis in mice brain Metabolic Brain Disease 37 7 2569 2579 35913570 10.1007/s11011-022-01007-3

[b13-tjb-48-06-432] Demircigil N Gul M Gokturk N Kustepe EK Bag HG 2023 Thymoquinone played a protective role against tartrazine-induced hepatotoxicity Iranian Journal of Basic Medical Sciences 26 1 99 106 10.22038/IJBMS.2022.67341.14763 36594061 PMC9790050

[b14-tjb-48-06-432] Fatemi I Khaluoi A Kaeidi A Shamsizadeh A Heydari S 2018 Protective effect of metformin on D-galactose-induced aging model in mice Iranian Journal of Basic Medical Sciences 21 1 19 10.22038/IJBMS.2017.24331.6071 29372032 PMC5776431

[b15-tjb-48-06-432] Gao Y Xu Y Yin J 2022 Selenomethionine ameliorates cognitive impairment, decreases hippocampal oxidative stress and attenuates dysbiosis in D-galactose-treated mice Antioxidants 11 1 111 10.3390/antiox11010111 35052615 PMC8772940

[b16-tjb-48-06-432] Grayson B Leger M Piercy C Adamson L Harte M 2015 Assessment of disease-related cognitive impairments using the novel object recognition (NOR) task in rodents Behavioural Brain Research 285 176 193 10.1016/j.bbr.2014.10.025 25447293

[b17-tjb-48-06-432] Hamdi A Viera-Alcaide I Costa S Lino-Neto T Guillén-Bejarano R 2023 A Sustainable Approach for the Valorization of Underutilized Date Fruits Molecules 28 15 5807 10.3390/molecules28155807 37570777 PMC10420846

[b18-tjb-48-06-432] Hong C Wang Z Zheng SL Hu WJ Wang SN 2023 Metrnl regulates cognitive dysfunction and hippocampal BDNF levels in D-galactose-induced aging mice Acta Pharmacologica Sinica 44 4 741 751 10.1038/s41401-022-01009-y 36229598 PMC10042843

[b19-tjb-48-06-432] Jeong HJ Yang SY Kim HY Kim NR Jang JB 2016 Chelidonic acid evokes antidepressant-like effect through the up-regulation of BDNF in forced swimming test Experimental Biology and Medicine 241 14 1559 1567 10.1177/1535370216642044 27037280 PMC4994898

[b20-tjb-48-06-432] Khairnar SI Kulkarni YA Singh K 2024 Mitigation of cisplatin-induced nephrotoxicity by chelidonic acid in Wistar rats Journal of Trace Elements in Medicine and Biology 81 127321 10.1016/j.jtemb.2023.127321 37918276

[b21-tjb-48-06-432] Koca RÖ 2019 Neurokinin 3 Receptor Effects on Cognitive Behaviour in a Rat Model of Alzheimer’s Disease Doctoral dissertation Necmettin Erbakan University Konya, Türkiye (in Turkish with an abstract in English)

[b22-tjb-48-06-432] Küçükkarapinar M Dönmez A Candansayar S Bozkurt A Akçay E 2021 Behavioral and Neurodevelopmental Effects of Early Interventions in Adult Wistar Rats Archives of Neuropsychiatry 58 2 137 10.29399/npa.24943 34188597 PMC8214749

[b23-tjb-48-06-432] Lee J Kim YS Kim E Kim Y Kim Y 2020 Curcumin and hesperetin attenuate D-galactose-induced brain senescence in vitro and in vivo Nutrition Research and Practice 14 5 438 10.4162/nrp.2020.14.5.438 33029285 PMC7520561

[b24-tjb-48-06-432] Li M Yang L Gao L Du G Qin X 2022 The leaves of Scutellaria baicalensis Georgi attenuate brain aging in D-galactose-induced rats via regulating glutamate metabolism and Nrf2 signaling pathway Experimental Gerontology 170 11 1978 10.1016/j.exger.2022.111978 36244586

[b25-tjb-48-06-432] Lian W Jia H Xu L Zhou W Kang D 2017 Multi-protection of DL0410 in ameliorating cognitive defects in D-galactose induced aging mice Frontiers in Aging Neuroscience 9 409 10.3389/fnagi.2017.00409 29276489 PMC5727065

[b26-tjb-48-06-432] Liao Y Lai Y Xu H Gao L Fu X 2023 Bushen-Yizhi formula ameliorates mitochondrial dysfunction and oxidative stress via AMPK/Sirt1 signaling pathway in D-gal-induced aging rats Chinese Medicine 18 1 53 10.1186/s13020-023-00755-3 37170155 PMC10176912

[b27-tjb-48-06-432] Lin B Xu D Wu S Qi S Xu Y 2021 Antioxidant effects of Sophora davidi (Franch.) Skeels on d–galactose–induced aging model in mice via activating the SIRT1/p53 pathway Frontiers in Pharmacology 12 754554 10.3389/fphar.2021.754554 34938181 PMC8687624

[b28-tjb-48-06-432] Lissner LJ Wartchow KM Toniazzo AP Gonçalves CA Rodrigues L 2021 Object recognition and Morris water maze to detect cognitive impairment from mild hippocampal damage in rats: A reflection based on the literature and experience Pharmacology Biochemistry and Behavior 210 173273 10.1016/j.pbb.2021.173273 34536480

[b29-tjb-48-06-432] Liu J Chen D Wang Z Chen C Ning D 2019 Protective effect of walnut on d-galactose-induced aging mouse model Food Science & Nutrition 7 3 969 976 10.1002/fsn3.907 30918639 PMC6418433

[b30-tjb-48-06-432] Lopez JAS González HM Léger GC 2019 Alzheimer’s disease Handbook of clinical neurology, 167 231 255 10.1016/B978-0-12-804766-8.00013-3 31753135

[b31-tjb-48-06-432] Miroshnichenko LA Polyakova TY Avdeeva EY Krivoshchekov SV Khlusov IA 2022 Chelidonic acid and its derivates: general spectrum of biological activity and osteogenic properties Drug Development & Registration 11 4 60 71 10.33380/2305-2066-2022-11-4-60-71

[b32-tjb-48-06-432] Mizoguchi Y Kitamura A Wake H Ishibashi H Watanabe M 2006 BDNF occludes GABAB receptor-mediated inhibition of GABA release in rat hippocampal CA1 pyramidal neurons European Journal of Neuroscience 24 8 2135 2144 10.1111/j.1460-9568.2006.05092.x 17074039

[b33-tjb-48-06-432] Nam SM Seo M Seo JS Rhim H Nahm SS 2019 Ascorbic acid mitigates D-galactose-induced brain aging by increasing hippocampal neurogenesis and improving memory function Nutrients 11 1 176 10.3390/nu11010176 30650605 PMC6356429

[b34-tjb-48-06-432] Porter TG Martin DL 1985 Chelidonic acid and other conformationally restricted substrate analogues as inhibitors of rat brain glutamate decarboxylase Biochemical pharmacology 34 23 4145 4150 10.1016/0006-2952(85)90207-2 4062982

[b35-tjb-48-06-432] Rehman SU Shah SA Ali T Chung JI Kim MO 2017 Anthocyanins reversed D-galactose-induced oxidative stress and neuroinflammation mediated cognitive impairment in adult rats Molecular Neurobiology 54 255 271 10.1007/s12035-015-9604-5 26738855

[b36-tjb-48-06-432] Shin HJ Kim HL Kim SJ Chung WS Kim SS 2011 Inhibitory effects of chelidonic acid on IL-6 production by blocking NF-κB and caspase-1 in HMC-1 cells Immunopharmacology and Immunotoxicology 33 4 614 619 10.3109/08923973.2011.552508 21320026

[b37-tjb-48-06-432] Singh DK Gulat K Ray A 2016 Effects of chelidonic acid, a secondary plant metabolite, on mast cell degranulation and adaptive immunity in rats International Immunopharmacology 40 229 234 10.1016/j.intimp.2016.08.009 27620504

[b38-tjb-48-06-432] Sumbalová Z Uličná O Kucharská J Rausová Z Vančová O 2022 D-galactose-induced aging in rats–The effect of metformin on bioenergetics of brain, skeletal muscle and liver Experimental Gerontology 163 111770 10.1016/j.exger.2022.111770 35314269

[b39-tjb-48-06-432] Sun K Yang P Zhao R Bai Y Guo Z 2018 Matrine attenuates D-galactose-induced aging-related behavior in mice via inhibition of cellular senescence and oxidative stress Oxidative Medicine and Cellular Longevit 10.1155/2018/7108604 PMC6288577 30598725

[b40-tjb-48-06-432] Tomoda T Sumitomo A Shukla R Hirota-Tsuyada Y Miyachi H 2022 BDNF controls GABAAR trafficking and related cognitive processes via autophagic regulation of p62 Neuropsychopharmacology 47 2 553 563 10.1038/s41386-021-01116-0 34341497 PMC8674239

[b41-tjb-48-06-432] Topuz RD 2015 Morris su labirenti uzaysal öğrenme ve bellek modelinde, sıçan hipokampüsündte histon asetilasyonu ve histon deasetilaz inhibitörünün etkisi Doctoral dissertation Trakya University Trakya, Türkiye (in Turkish)

[b42-tjb-48-06-432] Turan İ Özaçmak VH Özaçmak HS 2023 Melatonin Improves Postoperative Cognitive Dysfunction in Aged Rats: Relevance of Oxidative Stress, PSD95 and Ca2+/Calmoduline Dependent Protein Kinase Batı Karadeniz Tıp Dergisi 7 2 225 233 10.29058/mjwbs.1342979

[b43-tjb-48-06-432] Wu SC Lin CY Hong LJ Chen CC 2021 Automated Eight-Arm Maze Trajectory Tracking System for Feature Extraction of TBI Animals 2021 IEEE/ACIS 22nd International Conference on Software Engineering, Artificial Intelligence, Networking and Parallel/Distributed Computing Taichung, Taiwan 106 109 10.1109/SNPD51163.2021.9704966

[b44-tjb-48-06-432] Yin D Zhao L Deng S Xie Y Ro KS 2024 Lactiplantibacillus plantarum X7022 Plays Roles on Aging Mice with Memory Impairment Induced by D-Galactose Through Restoring Neuronal Damage, Relieving Inflammation and Oxidative Stress Probiotics and Antimicrobial Proteins 1 14 10.1007/s12602-023-10208-w 38183568

[b45-tjb-48-06-432] Yuan S Yang Y Li J Tan X Cao Y 2020 Ganoderma lucidum Rhodiola compound preparation prevent D-galactose-induced immune impairment and oxidative stress in aging rat model Scientific Reports 10 1 19244 10.1038/s41598-020-76249-1 33159105 PMC7648061

[b46-tjb-48-06-432] Yue J Guo P Jin Y Li M Hu X 2023 Momordica charantia polysaccharide ameliorates D-galactose-induced aging through the Nrf2/β-Catenin signaling pathway Metabolic Brain Disease 38 3 1067 1077 10.1007/s11011-022-01103-4 36287355

